# Commentary: Telemedicine for cancer patients in the COVIDian era—A long overdue promise?

**DOI:** 10.3389/fpubh.2022.931380

**Published:** 2022-08-18

**Authors:** Saravanan Sekaran, Parameswari Royapuram Parathasarathy, Sinduja Palati, Dhanraj Ganapathy, Khursheed Muzammil, Nazim Nasir

**Affiliations:** ^1^Department of Prosthodontics, Saveetha Dental College and Hospitals, Saveetha Institute for Medical and Technical Sciences, Chennai, Tamil Nadu, India; ^2^Department of Pharmacology, Centre for Transdisciplinary Research, Saveetha Dental College and Hospitals, Chennai, India; ^3^Department of Oral Pathology, Saveetha Dental College and Hospitals, Saveetha Institute for Medical and Technical Sciences, Chennai, Tamil Nadu, India; ^4^Department of Public Health, CAMS, Khamis Mushait Campus, King Khalid University, Abha, Saudi Arabia; ^5^Department of Basic Medical Sciences, CAMS, Khamis Mushait Campus, King Khalid University, Abha, Saudi Arabia

**Keywords:** COVID-19, telehealth, cancer, alternative, mobile

For the past 2 years, the COVID-19 pandemic has dramatically changed the cancer care landscape for social distancing. Limitations in all modes of transportation and lockdown rules had disrupted the cancer care nest by adversely delaying cancer diagnosis, hampering psychological support, and decreasing the pace of clinical trials. Accelerated adoption of telemedicine and telehealth strategies aided uninterrupted management and treatment of cancer patients globally. Cancer care is underlooked in patients with a reduction in appropriate care due to the distraction effect seen in hospitals handling COVID-19 patients (1). Telemedicine has emerged as not a mere interim solution but a strong and ideal tool to deliver cancer care to patients who are capable of adapting to this platform. Telemedicine has decreased the number of hospital visits by cancer patients who are vulnerable to COVID-19 infection and at high risk of death due to their suppressed immune system. The integrated function of telehealth depends on the selection of patients which is dependent on physicians' experience and case complexity. The European Society for Medical Oncology (ESMO) guidelines advises teleoncology services to patients on oral therapy for prescription renewal and follow-up consultation (2). It also recommends the use of teleservices for dose adaptation, toxicity evaluation, and imposing supportive care. EMSO instructs on a tiered approach for prioritizing and converting hospital visits into telemedicine for stable and non-critical care patients. The United States Drug Enforcement Administration permits palliative care professionals to provide opioid prescriptions to cancer patients through tele-oncology (3). Rapid technological advancements have led to the adoption of several technologies into telemedicine platforms by doctors, patients, health care workers, and regulatory bodies. Telemedicine has enabled real-time audio-visual interactions over in-person consultations for cancer patients (4).

We at our hospital see telemedicine as a very useful platform for delivering uninterrupted care to cancer patients from far and remote places during these COVID regulations. Discussion on cancer prognosis, treatment regimens, or recovery chances needs to be done in physical presence which would better convey the information. Telemedicine is not a perfect substitute for in-person clinical appointments, it would generally provide a false scenario of uncaring and insensitive clinicians to the patients. Therefore, we would like to take the liberty of conveying to the readers' various difficulties and solutions in adopting telemedicine as an alternative to in-person consultations for cancer care.

## Setbacks associated with telemedicine implementation

Telemedicine is rapidly adopted in oncology practice and management. Several healthcare workers also experienced clear benefits of imparting telemedicine such as privacy, a streamlined system, and reduced wait times. However, the feasibility of telemedicine across the globe especially for people from rural areas is under debate and equitable implementation requires addressing several challenges mentioned below:

1. Insufficient exposure and training to telemedicine technology and limited physical examination sessions are among the major setbacks of telemedicine. What is more important to consider is the ability of the patients to learn and use such technologies.
2. Telemedicine demands experience in internet-based applications. The majority of the cancer patients are of the older population and/or dwell in remote areas, the accessibility to telemedicine is limited due to poor internet access. For instance, over 25,000 villages do not have cellular or internet services and lack network connectivity (5).
3. Accurate diagnosis over telehealth is often troublesome. The lack of interpersonal connection reduces the effectiveness of optimal care where non-verbal communication and body language expression is missing to indicate psychological distress.
4. Telemedicine is certainly impractical in cancer patients with visual impairment, hearing loss, and cognitive difficulties.
5. Patients also experience nervousness and anxiety in using video-enabled platforms which often decreases the communication with the clinicians which would miss addressing vital complaints from patients.
6. Lack of proper guidelines and management merely based on only symptoms and elusiveness of symptoms in patients with head and neck cancers would often affect the treatment plan.
7. Most of the cancer specialists and cancer care centers are more populated in cities compared to rural areas. As telemedicine could bridge the gap and help patients from several places, the digital divide is seen in patients from rural backgrounds and cities in low- and middle-income countries, limiting access to telemedicine.
8. The informed choice of agreeing to telemedicine across patients from different ethnical backgrounds is largely variable. They experience stress and are reluctant in communicating through internet platforms (especially video conferencing).
9. The diagnosis and follow-up of oral lesions on video calls and photographs are difficult as most of the oral lesions both cancerous and potentially cancerous are more often seen in the posterior areas.
10. Financial constraints in patients with low income limit the reach of telemedicine over in-person consultations. A significant population of people still lack the use of sophisticated mobile devices to handle e-health apps.
11. Finally, telemedicine needs to be performed over a secure, trusted end-to-end encrypted network to ensure data integrity and protection against leaks. Professionals and patients' need to be properly guided with appropriate mobile applications to be used for telemedicine. The lack of trusted free applications and paid applications decrease the pace of telemedicine reach to the public.

## Recommended solutions for the obstacles witnessed in telemedicine implementation

Empowering patients to use telemedicine services is very vital in witnessing its reach in the future. Enabling equity in access to health care through telemedicine can be facilitated by creating brochures/handouts and television programs with standard operating procedures and protocols for telemedicine. Besides health illiteracy prevailing across various nations, patients can send images of prescriptions and laboratory reports to clinicians. The process can be enhanced through empowerment sessions for the patients. Providing cheap internet services, enhancing internet connectivity even in remote areas, budget-friendly mobile phones, and operating awareness will definitely improve the reach and implementation of telemedicine services. To ascertain societal needs and facilitate engagement of users in telemedicine among various ethnicity, connecting educators, community leaders, academicians, health administrators, healthcare professionals, and policymakers must be effectively initiated. Apart from this, regulatory frameworks and laws governing telemedicine use are essential to address concerns on data privacy and patient consent procedures.

## Future directions

One comprehensive question about telemedicine implementation is whether this is a temporary adjustment to the conventional clinical practice or a sustained strategy even after the COVIDian era. What is clear about telemedicine in cancer care is its inability to address critical concerns owing to patient compliance and palliative care. We should hope to preserve the option of adopting telemedicine for appropriate patients who are clinically stable. It will definitely not be a substitute for conventional clinical care but could remain connected as a hybrid mode along with standard cancer care. The immature practice of telemedicine is expected to grow and its catalyzation in the forthcoming years would provide comprehensive care for cancer patients.

## Author contributions

SS, PP, SP, DG, KM, and NN collected literature and drafted the manuscript. KM and NN provided technical help and secured funding. SS designed the manuscript and approved the final submitted manuscript. All authors contributed to the article, during revision and approved the submitted version.

## Conflict of interest

The authors declare that the research was conducted in the absence of any commercial or financial relationships that could be construed as a potential conflict of interest.

## Publisher's note

All claims expressed in this article are solely those of the authors and do not necessarily represent those of their affiliated organizations, or those of the publisher, the editors and the reviewers. Any product that may be evaluated in this article, or claim that may be made by its manufacturer, is not guaranteed or endorsed by the publisher.
